# Novel insights into extrachromosomal DNA: redefining the onco-drivers of tumor progression

**DOI:** 10.1186/s13046-020-01726-4

**Published:** 2020-10-12

**Authors:** Xiang Gu, Jie Yu, Peiwei Chai, Shengfang Ge, Xianqun Fan

**Affiliations:** 1 Department of Ophthalmology, Ninth People’s Hospital, Shanghai JiaoTong University School of Medicine, Shanghai, 20025 P. R. China; 2 Shanghai Key Laboratory of Orbital Diseases and Ocular Oncology, Shanghai, 20025 People’s Republic of China

**Keywords:** Extrachromosomal DNA, Oncogene, Tumorigenesis

## Abstract

Extrachromosomal DNA (ecDNA), gene-encoding extrachromosomal particles of DNA, is often present in tumor cells. Recent studies have revealed that oncogene amplification via ecDNA is widespread across a diverse range of cancers. ecDNA is involved in increasing tumor heterogeneity, reverting tumor phenotypes, and enhancing gene expression and tumor resistance to chemotherapy, indicating that it plays a significant role in tumorigenesis. In this review, we summarize the characteristics and genesis of ecDNA, connect these characteristics with their concomitant influences on tumorigenesis, enumerate the oncogenes encoded by ecDNA in multiple cancers, elaborate the roles of ecDNA in tumor pathogenesis and progression, and propose the considerable research and therapeutic prospects of ecDNA in cancer.

## Background

Genetic material guarantees the transmission of genetic information from parents to their offspring. With the exception of some viruses, DNA is the carrier of genetic material, which was first identified by Frederick Griffith in a *Streptococcus pneumoniae* transformation experiment [1–3]. DNA is present in a compacted and dynamic complex called chromatin in the cell nucleus. During the metaphase of cell division, chromatin organizes into highly condensed chromosomes [4]. DNA also exists in organelles including mitochondria and chloroplasts, which probably evolved from microorganisms such as α-proteobacteria and cyanobacterium invading host cells and starting a symbiotic life form [5–8]. In addition, it has been reported that extrachromosomal particles of DNA exist, the size of which varies from dozens to millions of base pairs [9, 10]. These particles have been proved to be circular by biophysical methods and DNA sequencing. Verhaak et al. divided these particles into two types based on the size and resultant functions [11]. Small particles, which are called extrachromosomal circular DNA (eccDNA), are usually less than 1 kb; consequently, they are undetectable by light microscopy and lack full-length genes [12–14]. Investigations of eccDNA have revealed its association with aging, as eccDNA containing ribosomal DNA genes accumulates in old cells and contributes to the aging of yeast cells. The probable mechanism is described by the titration hypothesis, which indicates that the accumulated eccDNA might precipitate the components of replication and/or transcription elements, and eventually trigger the senescence and the eventual death of old cells [15, 16]. Cohen et al. revealed that small polydispersed circular DNA (spcDNA), one type of eccDNA, is related to chromosomal instability (CIN), which is a characteristic of malignant cells since the spcDNA molecules were frequently found in either intrinsically unstable cells including tumor cells or in cells exposed to an external carcinogen treatment, indicating that spcDNA may be a possible marker of CIN [17, 18]. Extrachromosomal DNA (ecDNA), the focus of this review, includes relatively large particles generally ranging from 1 to 3 Mb, with a median size of 1.26 Mb, which are therefore visible by light microscopy and encompass whole genes as well as regulatory regions [11, 19–21]. ecDNA was first discovered as paired small chromatin bodies in neuroblastoma (NB) cells called double minutes (DMs) [10]. Jack et al. performed a scanning electron microscopy (SEM) study of DMs and demonstrated that the identical sister minutes appeared as two spherical chromatin bodies interconnected by chromatin fibers [22]. Later, Turner et al. performed whole-genome sequencing (WGS), structural modeling and cytogenetic analyses of 17 different cancer types and found that only 30% of ecDNA in tumor cells is paired; thus, ecDNA is used as a general term to refer to large, gene-containing extrachromosomal particles of DNA, including both DMs and single body forms, with single minutes showing no apparent partners nearby. The existence of ecDNA has been confirmed in various types of cancers and cancer cell lines [23]. Unlike the smaller eccDNA particles, ecDNA is sufficiently large enough to contain one or multiple full genes, including oncogenes, and oncogenes located on ecDNA were first reported to map *MYCN* to DMs in NB [24]. Subsequent studies confirmed the existence of oncogene amplification on ecDNA in multiple types of cancer [25–28]. Although oncogene amplification via ecDNA has been recognized, ecDNA was thought to be rare in cancer for a long time; therefore, the role of ecDNA in tumorigenesis has received little attention [29, 30]. This perception was maintained until an unbiased approach was developed to detect ecDNA by integrating WGS and cytogenetic image analysis. The results showed that oncogene amplification via ecDNA is widespread in different cancers, thus arousing people’s interest in extrachromosomal oncogene amplification when exploring tumorigenesis [23]. In this review, we focus on the characteristics and genesis of ecDNA and emphasize its role in tumorigenesis.

## Characteristics of ecDNA

The rapid development of new techniques enables determination of the unique characteristics of ecDNA. Compared to chrDNA, ecDNA is acentric with uneven segregation at cell division, leading to increased intratumoral heterogeneity and enhanced fitness to changing environments. In addition, ecDNA can be eliminated via micronucleus formation to reverse the tumor phenotype. Furthermore, ecDNA is circular with high chromatin accessibility and ultra-long-range chromatin contact. We will summarize the unique characteristics of ecDNA and resultant outcomes in cancer (Table 1).

**Table 1:** The unique characteristics of ecDNA compared to chrDNA and their outcomes in tumorigenesis

	chrDNA	ecDNA	Function of ecDNA based on the difference	Outcome in tumorigenesis	Reference
Structure	Circular	Linear	High chromatin accessibility and ultra-long-range chromatin contact	High levels of oncogene transcription and expression	[19]
Centromere	Centric	Acentric	Uneven segregation during cell division	Increased intratumoral heterogeneity and enhanced fitness to changing environments	[23, 31–33]
Stability	Stable	Unstable	Eliminable via micronucleus formation	Tumor phenotype reversion	[34–39]

### ecDNA is acentric with uneven segregation at cell division

The centromere, which consists of repetitive DNA, is the region where sister chromatids remain connected until mitosis and has a unique histone—CenpA. The centromere is the ‘landing platform’ for the kinetochore, a large protein structure that binds to microtubules in mitosis and ensures accurate sister chromatid separation and chromosome segregation [40, 41]. Because centromeres lack ecDNA, ecDNA segregates unevenly at cell division, allowing daughter cells to possess up to twice as many ecDNA particles as their corresponding mother cells [31]. Uneven segregation of ecDNA leads to genetic material distribution uncertainty, which may result in a rapid increase in oncogene copy number in a short period and increase intratumoral heterogeneity, enhancing tumor fitness to changing environments [32].

### The resultant heterogeneity in cancer with ecDNA

deCarvalho et al. revealed that ecDNA presented divergent inheritance patterns and clonal selection dynamics through an extensive genomic and transcriptomic analysis of 13 glioblastoma (GBM) tumor samples and neurosphere-forming cultures and orthotopic xenograft models established from these samples, inferring the uneven inheritance of ecDNA between offspring cells, which may explain the rapidly enhanced genomic heterogeneity during GBM evolution [42]. Xu et al. characterized DMs in paired diagnosis and relapse tumors from 4 GBM patients and found that identical oncogenes could amplify on different DMs at diagnosis and relapse, reflecting the primed evolution and rapidly increasing heterogeneity of DMs [43].

### ecDNA can be eliminated by micronucleus formation

**Fig. 1:**
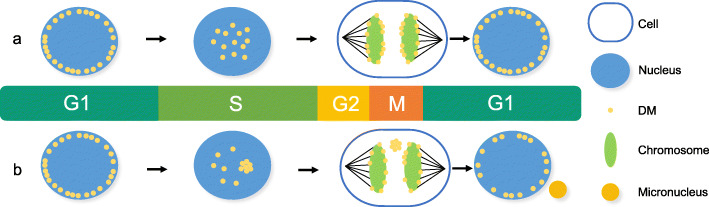
Generation of DM-type micronuclei. **a**. DMs are located on the nuclear periphery during the G1 phase and move to the interior during their replication in the early S phase. Acentric DMs segregate by adhering to the chromosome arm during the M phase, which is called ‘hitchhiking.’ **b**. In cells exposed to a low dose of HU during the early S phase, DMs tend to aggregate and detach from the chromosome during the subsequent M phase. Then, the aggregated DMs generate micronuclei during the subsequent interphase

Oncogenes on DMs are unstable, and DMs can be eliminated in tumor cells by incorporating DMs into the cytoplasmic micronucleus when they are exposed to DNA replication inhibitors such as a low dose of hydroxyurea (HU) and radiation [34, 35]. In addition, inhibition of ERK1/2 activation, one of the main mitogen-activated protein kinase (MAPK) signaling pathways, also breaks DNA to eliminate DMs [36]. Acentric DMs were found to segregate by adhering to the chromosome arm during cell division, a process is called ‘hitchhiking’ (Fig. 1.a). When cells are exposed to a low dose of HU, DMs aggregate and detach from the chromosome; subsequently, the aggregated DMs generate micronuclei (Fig. 1.b). The micronuclei may be eliminated, religated, or integrated into the main nucleus or generate giant DMs or homogeneously staining regions (HSRs) [37]. Since a decrease in oncogene amplification in tumor cells to revert the tumor phenotype has been reported in various cancers [44–49], elimination and morphological transformation of DMs may result in a reduction in tumorigenicity and a loss of malignant properties. However, HU treatment does not decrease the copy number of oncogenes amplified on HSRs, another form of oncogene on chromosomes, indicating that oncogene amplification is unstable on ecDNA but stable on HSRs [38]. In addition to being eliminated by DNA replication inhibitors, DMs can be eliminated by suppressing the expression of genes residing on DMs. Suppression decreases the number of DMs and reduces oncogene amplification on DMs through micronucleus formation, resulting in a reduction in tumor proliferation and invasion [39].

### The resultant tumor reversion in cancer with ecDNA

Ambros et al. used fluorescence in situ hybridization (FISH) with a *MYCN*-specific probe in NB cell lines and revealed spontaneous elimination of extrachromosomally amplified *MYCN* in F-cells with significant biological features and loss of the malignant phenotype and properties. Therefore, elimination of extrachromosomally amplified *MYCN* in NB is postulated to be associated with tumor cell reversion [46].

### ecDNA is circular with highly accessible chromatin and ultra-long-range chromatin contacts

**Fig. 2:**
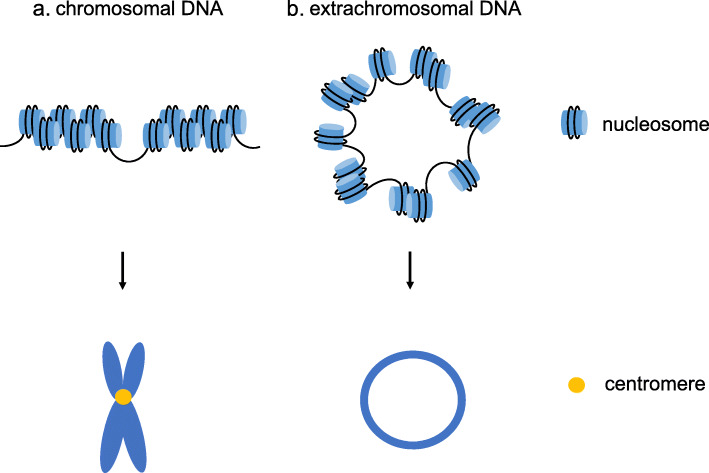
Structures of ecDNA and chrDNA. **a**. The nucleosomal organization of chrDNA is compact, and during cell division, DNA is packaged into chromosomes with centromeres. **b**. The nucleosomal organization of ecDNA is less compact than that of chrDNA, and ecDNA is circular without centromeres

Based on biophysical methods and DNA sequencing, ecDNA is thought to be circular [50]. Recently, the distinct structure of ecDNA was demonstrated to be circular through the use of ultrastructural imaging, computational WGS analyses, long-range optical mapping and superresolution three-dimensional structured illumination microscopy (3D-SIM), scanning electron microscopy (SEM) and transmission electron microscopy (TEM). ecDNA is packaged into chromatin with nucleosome units, which mainly contain active histone marks, and its chromatin landscape is more accessible than that of chromosomal DNA (chrDNA) due to its nucleosomal organization being less compact than that of chrDNA (Fig. 2). Since the number of transcripts is markedly increased when oncogenes are present in circular ecDNA, even after normalization for the DNA copy number, the transcription levels are still high, indicating that the active chromatin state and highly accessible chromatin may be associated with the high transcription level of ecDNA. Moreover, ultra-long-range chromatin contact can occur on ecDNA, revealing the potential influence of ecDNA on distal gene expression [19].

### The resultant distal chromatin contact in cancer with ecDNA

Morton et al. resolved *EGFR* ecDNA amplicon patterns from primary GBM sequencing and discovered that amplified noncoding regions are inclined to incorporate enhancers and that ecDNA structures always include adjacent enhancers, indicating that enhancers are co-selected with the coding regions of oncogenes during the formation of ecDNA. Furthermore, they revealed that the *EGFR* locus amplified upon ecDNA contact with the distal enhancer by chromosome conformation experiments and confirmed that these co-selected enhancers are partly outside the topologically associated domain (TAD) of the original chromosome [51].

## Genesis of ecDNA

**Fig. 3:**
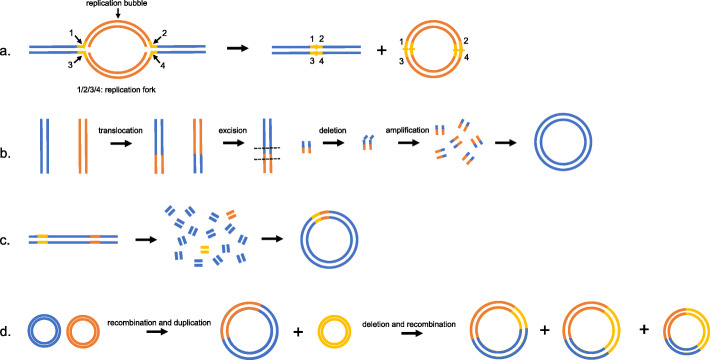
Models of the possible ecDNA genesis mechanism. **a**. ‘Episome’ model. Bidirectional replication of DNA leads to the existence of two replication forks, and the region between them is named the replication bubble. When an error in replication occurs, the replication forks arrest, and the replication bubble drops and transforms into an episome, which further replicates and recombines to form DMs. **b**. ‘Translocation-excision-deletion-amplification’ model. Two chromosomes translocate before excision and deletion occur in close proximity to the translocation breakpoints. Then, amplification and circularization of the separate fragments lead to the formation of DMs. **c**. ‘Chromothripsis’ model. The mis-segregation of a chromosome results in the chromosome shattering and subsequent random repair to form DMs, allowing non-contiguous DNA fragments to fuse in the DMs. **d**. A multistep evolutionary process in the GLC1 cell line. Recombination and duplication of two single-chromosomal ancestral DMs can occur first, and then the new DMs undergo various types of deletion and recombination with another DM of a single-chromosomal source, resulting in multiple DM subpopulations

Although the precise ecDNA genesis mechanism is still unknown, some models have been established to suggest the possible mechanism, including the episome model, the translocation-excision-deletion-amplification model, chromothripsis and a multistep evolutionary process (Fig. 3 and Table 2).

**Table 2:** The mechanism of ecDNA genesis

Model	Mechanism	Reference
Episome	Replication bubble drop from the chromosome to produce an episome and then the episome replicates and recombines to form ecDNA.	[52–55]
Translocation-excision-deletion-amplification	Two chromosomes translocate with subsequent excision and deletion at the regions neighboring translocation breakpoints, and then the separate fragments amplify and circularize to form ecDNA.	[56]
Chromothripsis	Chromosomes are isolated into fragments through an error in cell segregation, and the fragments connect randomly to produce ecDNA.	[57–59]
A multistep evolutionary process	The single-chromosome ancestral episomes undergo a series of discrete mutation events, including recombinations, deletions and duplications to produce various ecDNAs.	[60, 61]

### Episome in ecDNA

One of the classic models is the episome model, where replication fork stalling elicits replication fork collapse, and the replication bubble subsequently falls off the chromosome, interconnects and transforms into an extrachromosomal circular molecule named the episome, with autonomous replication and recombination of episomes leading to the formation of DMs (Fig. 3.a) [52, 53]. The definitive evidence underpinning the episome model is the genesis of *MYC*-containing DMs in acute myeloid leukemia (AML). Storlazzi performed FISH analysis on metaphase chromosomes of bone marrow samples derived from the 30 AML patients with *MYC*-containing DMs and discovered that 23 samples showed a deletion of the *MYC* gene region on chromosome 8. DNA sequencing revealed that the linker sequences of DMs were as the same as the linker sequences of chromosome 8 with a deletion of the *MYC* gene region, indicating that the gene carried by DMs originated from chromosome 8, which is consistent with the episome model [54]. Later, the episome model was suggested to also be applicable to solid tumors, including NB and small cell lung cancer (SCLC) [55].

### Translocation-excision-deletion-amplification in ecDNA

Van Roy et al. discovered the co-localization of *MYC* and *ATBF1* on the same DMs in the NB cell line SJNB-12 and proposed another model called translocation-excision-deletion-amplification. The model was prompted by the reciprocal translocation between chromosomes 8 and 16 on which *MYC* and *ATBF1* were located, with subsequent excisions and deletions occurring in close proximity to the translocation breakpoints, and then the isolated sequence amplified and circularized to form DMs (Fig. 3.b).

### Chromothripsis in ecDNA

Cancer genome sequencing identified a new model called chromothripsis in which a chromosome mis-segregates and ends up in a micronucleus where it has less access to replication proteins, leading to replication stress, chromosome breaks and subsequent faulty repair (Fig. 3.c) [57, 58]. The model was supported by a xenografted human oligodendroglioma where co-amplification of *EGFR* and *MYC* existed in the form of DMs. Vogt et al. discovered that the small fragments localized hundreds of kilobases apart on the chromosome were associated with contigs in the DMs, and that the various junctions were associated with fusions between non-contiguous sequences in the normal reference genome through FISH and WGS, indicating that the presence of rearrangements during the formation of DMs is related to the chromothripsis model [59].

### A multistep evolutionary process in ecDNA

L’Abbate et al. investigated the SCLC cell line GLC1 through an integrated approach combining next-generation sequencing technologies (NGS) and single-nucleotide polymorphism (SNP) array, polymerase chain reaction (PCR) and FISH techniques and proposed the presence of multiple DM subpopulations with the variety of shared structural variations (SVs), with some consisting of a single chromosome and others carrying combinations of different chromosomes, suggesting that a multistep evolutionary process starting from single-chromosome ancestral DMs can better explain processes involving more than two chromosomes. For example, in the GLC1 cell line, single-chromosome ancestral DMs derived from chromosome 1, 8 or 21 undergo a series of mutation events, including recombinations, deletions and duplications, to transform into multiple DM subpopulations. The ancestral DMs derived from chromosomes 1 and 8 recombined and duplicated first to form a new DM, and then various types of deletion and recombination events occurred between the new DM and ancestral DM derived from chromosome 21, leading to multiple DM subpopulations (Fig. 3.d) [62, 63].

## ecDNA in cancer

**Fig. 4:**
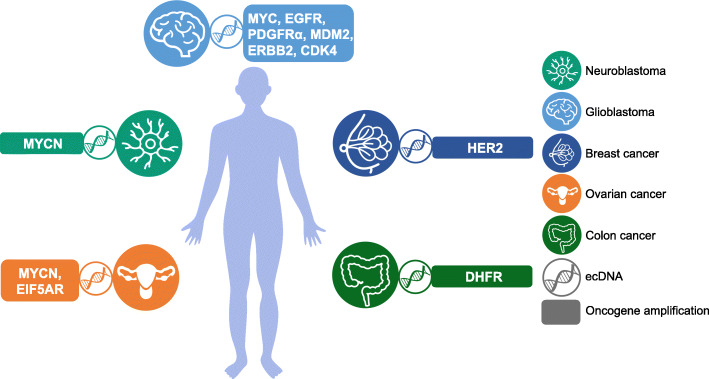
ecDNA-mediated oncogene amplification promotes tumor progression. In NB, *MYCN* encoded by ecDNA is closely associated with tumor progression. In glioblastoma, the amplification of oncogenes, such as *MYC*, *EGFR*, *PDGFRα*, *ERBB2*, *CDK4*, and *MDM2*, on ecDNA promotes tumorigenesis. In breast cancer, *HER2* encoded on ecDNA contributes to tumorigenesis. In ovarian cancer, ecDNA containing *MYCN* and *EIF5AR* stimulates tumorigenesis. In colon cancer, *DHFR* amplification via ecDNA is also involved in tumorigenesis

ecDNA is abundant in cancer cells but rare in normal cells, and the copy number of ecDNA varies in different types of cancer, with the highest prevalence in GBM [23]. Notably, ecDNA is a vehicle for oncogene amplification, and the role of oncogenes in tumorigenesis is well studied in vitro and in vivo, indicating that ecDNA plays a considerable role in tumorigenesis. We will review recent reports describing our understanding of the biological functions of ecDNA in various types of cancer and explore new mechanisms for tumor pathogenesis and evolution (Fig. 4 and Table 3).

**Table 3:** The roles of ecDNA in tumorigenesis

Cancer	Oncogene amplification via ecDNA	The connection between ecDNA and tumorigenesis	Reference
Neuroblastoma	*MYCN*	ecDNA remodels the chromosomal genome and affects chromosomal gene expression, including oncogenes and tumor suppressors	[64]
Glioblastoma	*MYC, EGFR, PDGFRα, ERBB2, CDK4, MDM2*	The oncogenes residing on ecDNA are dynamically regulated to evade therapy	[63, 65]
Colon cancer	*DHFR*	Tumor cells upregulate *DHFR* amplification via ecDNA to develop drug resistance	[66–68]
Ovarian cancer	*MYCN, EIF5AR*	The noncoding regions of ecDNA may regulate gene expression	[69]
Breast cancer	*HER2*	Dynamic regulation of oncogenes residing on ecDNA is not supported by *HER2*	[70]

### ecDNA in neuroblastoma

ecDNA was first discovered in metaphase spreads of NB cells [10]. In addition, Alt et al. used cloning methods to demonstrate that a new oncogene *MYCN* was mapped to DMs in the human NB cell line IMR-32 [24]; this is the first report confirming the presence of oncogenes in ecDNA. Ambros et al. investigated the NB cell lines with DMs in detail and discovered that when exposed to low-dose HU, the cells manifested an enlarged and flattened morphology and increased granularity and expressed senescence-associated-β-galactosidase (SA-β-GAL), all of which are features of cell senescence, indicating that low-dose HU is an effective senescence activator for tumor cells with DMs [71]. A recent study revealed that circular DNAs, including ecDNA, eccDNA and neochromosomes, unexpectedly remodel the chromosomal genome. The remodeling arises from the generation of circular DNA with the subsequent reintegration into chromosomal genomic loci, affecting chromosomal gene expression, including aberrant expression of oncogenes and tumor suppressors. Specifically, the adjacent circle integration of the oncogene TERT is associated with its augmented expression, and the integration of circle fragments into the tumor suppressor DCLK1 is associated with its repressed expression. Moreover, Kaplan–Meier analysis comparing the survival of 22 NB patients with circle-derived remodeling to that of 59 NB patients lacking such remodeling revealed that circle-derived remodeling was associated with adverse outcomes, which may explain the clinical heterogeneity observed in NBs [66].

### ecDNA in glioblastoma

Zhou et al. used two syngeneic primary cultures of GBM that differed in the presence or absence of *EGFR*-encoding DMs and found that the former had higher levels of invasiveness, heterogeneity and radioresistance. However, whether the presence of *EGFR*-coding DMs or upregulation of *EGFR* leads to these observations requires further research [62]. Nathanson et al. revealed the reversible suppression of ecDNA containing a functional mutation in epidermal growth factor receptor vIII (*EGFRvIII*) to evade targeted therapy—epidermal growth factor receptor (EGFR) inhibitors. Specifically, they discovered an unexpected phenomenon in which EGFR inhibitors decreased the number of tumor cells with high levels of *EGFRvIII* in vitro and in vivo, and after drug removal, the tumor cells containing a high copy number of *EGFRvIII* returned, which exceeded the classical genetics. Then, through observation of cells in metaphase, they found the possible mechanism through which *EGFRvIII* almost completely amplified on ecDNA; moreover, the number of ecDNAs containing *EGFRvIII* is reduced by EGFR inhibitors and then reemerged 1–2 weeks after drug removal [63]. Later, Nikolaev et al. developed a model called amplification-linked extrachromosomal mutations (ALEMs) to explain the above observation. ALEMs are a novel type of cancer variant originating from the extrachromosomal region and can be eliminated from cancer cells. The model is based on the fact that proliferation-promoting oncogenes reside on ecDNA, and ecDNA increases the mutational possibility. ALEMs occur not only in *EGFR* but also in *PDGFRα* and *ERBB2* according to exome sequencing of 7 GBM patients. Moreover, an analysis of 4198 tumors indicated the presence of ALEMs in various tumor types, indicating that ALEMs may be the foundation of resistance to therapies in multiple types of tumors [65]. The enhancers in the noncoding regions, which are co-selected with the coding regions of oncogene *EGFR* during the formation of ecDNA, are outside the TAD of the original chromosome, which may reflect the ability of ultra-long-range chromatin contacts in ecDNA. Moreover, by ablating individual enhancer activity by CRISPR interference, these enhancers are rewired in ecDNA to enhance oncogene *EGFR* expression and tumor fitness, suggesting a novel mechanism of enhancers in regulating oncogene amplification [51].

### ecDNA in colon cancer

Gene amplification is a frequent manifestation of genomic instability in human tumors and plays a critical role in tumor progression and drug resistance [72]. Methotrexate (MTX) resistance through dihydrofolate reductase (*DHFR*) gene amplification is the most common mechanism of drug resistance [71]. Morales et al. demonstrated that in colon cancer HT29 cells with a high MTX dose, the *DHFR* gene copy number is markedly increased via extrachromosomal DMs. Furthermore, they discovered that the loss of the *DHFR* amplicon occurs contemporaneously with the withdrawal of MTX in MTX-sensitive cells, and these cells present a decreased drug resistance capacity when they are re-exposed to MTX, providing a potential second-round treatment option for patients with drug resistance occurring via gene amplification [66]. Moreover, Meng et al. discovered that nonhomologous end joining (NHEJ) plays an important role in the formation of DMs; therefore, depletion of DNA-PKc, a key NHEJ protein, decreases the amplification of DHFR, resulting in increased MTX sensitivity. This discovery suggests that NHEJ may be targeted for the treatment of MTX-resistant colon cancer [67]. Subsequently, the same authors investigated the function of homologous recombination, another DSB repair pathway involved in gene amplification, and found that in comparison to MTX-sensitive cells, MTX-resistant cells present increased homologous recombination activity. Silencing *BRCA1*, a key player in homologous recombination, decreased the number of DMs and the copy number of oncogenes amplified on DMs, and the sensitivity to MTX increased in DM-containing MTX-resistant cells. However, *BRCA1* silencing had no influence on the copy number of oncogenes amplifying on HSRs and had no effect on the sensitivity to MTX in HSR-containing MTX-resistant cells; these findings indicate that the homologous recombination pathway may also be a target for the improvement of chemotherapeutic effects by decreasing extrachromosomal oncogene amplification [68].

### ecDNA in ovarian cancer

Jin et al. proposed the considerable effect of noncoding regions on DMs. They discovered 5 matrix attachment regions (MARs) in a 682 kb DM from human ovarian cancer cell line UACC-1598 by sequencing and bioinformatics analyses and determined that these MARs can interact with the nuclear matrix both in vitro and in vivo through electrophoretic mobility shift assay (EMSA) and PCR. Moreover, the transfection of MARs constructs into human embryonic kidney 293 T cells revealed the upregulation of oncogenes *MYCN* and *EIF5A2*, which localized near MARs, indicating that noncoding regions on DMs may regulate gene expression and play a crucial role in oncogene activation [69].

### ecDNA in breast cancer

Approximately 20% of breast cancers demonstrate amplification of the proto-oncogene *HER2*, a receptor tyrosine kinase (RTK) belonging to the epidermal growth factor receptor (EGFR) family, and approximately 30% of *HER2*-positive tumors show DM amplification [70, 73]. Although these tumors can respond to therapies against *HER2* directly, they often develop resistance and resume their progression [74]. In various models with resistance to anti-*HER2* therapy, the number of DMs containing *HER2* has been shown to be preserved, even when the resistance is acquired by the loss of HER2 protein expression, suggesting that the loss of HER2 protein expression due to the loss of DMs containing *HER2* is not a likely mechanism underlying resistance to anti-*HER2* therapy [70].

## Conclusion and perspectives

Recent years have witnessed tremendous advancements in the field of ecDNA. A wide array of oncogene amplification via ecDNA has been uncovered, reverting the concentration of research on the association between ecDNA and tumorigenesis [23]. However, the understanding of ecDNA and its influence on tumors remains limited. Fortunately, the rapid development of biotechnology has enabled various methods to be used to detect and construct ecDNA, offering an opportunity to confirm the structure of ecDNA and speculate its role in tumorigenesis [19].

Notably, many studies have revealed the features of ecDNA and proposed that ecDNA plays a considerable role in tumorigenesis, including NB, GBM, colon cancer, ovarian cancer and breast cancer [64, 67, 69, 75–77]. The elimination of ecDNA is confirmed and it can decrease oncogene amplification on ecDNA to revert the tumor phenotype [34, 37, 78]. Although the current methods for ecDNA elimination lack specificity, ecDNA is expected to be a good therapeutic target in the future. Circular and acentric ecDNA contributes to increased oncogene copy number and tumor heterogeneity, thus providing tumor cells with the ability to respond rapidly to changing environments, including treatment [19, 79]. A contradictory finding is that tumor cells can develop drug resistance through the increased copy number of oncogenes on ecDNA, such as *DHFR*, while resistance can also be acquired through the downregulation of the expression of oncogenes on ecDNA, such as *EGFR*, indicating that different methods exist to evade treatment [63, 66]. However, the mechanisms underlying these different methods are unknown. Tumor cells are hypothesized to tend to have a survival advantage according to the specific environment, called “passive choice”. Another possible mechanism is that tumor cells perceive the environment and then change the quantity of ecDNA that they carry to fit the environment, called “initiative regulation”. Understanding the underlying molecular mechanism of ecDNA in evading therapy is essential for exploring novel therapies.

The perception that ecDNA possesses a unique structure and function, the discovery that ecDNA drives oncogene amplification and the profound significance of ecDNA in cancer increase the understanding of current cancer genome maps, rendering ecDNA a hotspot for the investigation of tumor pathogenesis and evolution. To develop novel tumor therapies, numerous endeavors are required for a thorough understanding of ecDNA. Knowledge of the precise mechanism of the formation, maintenance, replication and influence of ecDNA in tumorigenesis merely represent the tip of the iceberg, offering a direction for future exploration, and continuous advancement of technology and unremitting exploration are necessary to eventually unveil the mysteries of ecDNA.

## Data Availability

Not applicable.

## Abbreviations

3D-SIM: Three-dimensional structured illumination microscopy
ALEMs: Amplification-linked extrachromosomal mutations
AML: Acute myeloid leukemia
chrDNA: Chromosomal DNA
CIN: Chromosomal instability
Circle-seq: Circle-sequencing
*DHFR*: Dihydrofolate reductase
DMs: Double minutes
DSBs: Double-strand breaks
eccDNA: Extrachromosomal circular DNA
ecDNA: Extrachromosomal DNA
*EGFR*: Epidermal growth factor receptor
*EGFRvIII*: Epidermal growth factor receptor vIII
EMSA: Electrophoretic mobility shift assay
FISH: Fluorescence in situ hybridization
GBM: Glioblastoma
HSRs: Homogeneously staining regions
HU: Hydroxyurea
MAPK: Mitogen-activated protein kinase
MARs: Matrix attachment regions
MTX: Methotrexate
NB: Neuroblastoma
NGS: Next generation sequencing technologies
NHEJ: Nonhomologous end joining
PCR: Polymerase chain reaction
RTK: Receptor tyrosine kinase
SA-β-GAL: Senescence-associated-β-galactosidase
SCLC: Small cell lung carcinoma
SEM: Scanning electron microscopy
SNP: Single nucleotide polymorphism
spcDNA: Small polydispersed circular DNA
SVs: Structural variations
TAD: Topologically associated domain
TEM: Transmission electron microscopy
TKIs: Tyrosine kinase inhibitors
WGS: Whole-genome sequencing
